# Women’s sexual autonomy as a determinant of cervical cancer screening uptake in Addis Ababa, Ethiopia: a case–control study

**DOI:** 10.1186/s12905-022-01829-4

**Published:** 2022-06-17

**Authors:** Miresa Midaksa, Alemnew Destaw, Adamu Addissie, Eva Johanna Kantelhardt, Muluken Gizaw

**Affiliations:** 1 Addis Ababa Food, Medicine and Health Care Administration and Control Authority, Addis Ababa, Ethiopia; 2 Department of Epidemiology and Biostatistics, School of Public Health, College of Medicine and Health Science, Mizan-Tepi University, Mizan-Teferi, Ethiopia; 3 Department of Preventive Medicine, School of Public Health, Addis Ababa University, Addis Ababa, Ethiopia; 4 Institute for Medical Epidemiology, Biometrics and Informatics Martin-Luther-University, Halle-Wittenberg, Germany; 5 Department of Gynecology, Martin-Luther-University, Halle-Wittenberg, Germany

**Keywords:** Cervical cancer, Screening, Determinants, Sexual autonomy

## Abstract

Cervical cancer (CC) is the fourth most common cancer in women worldwide and the leading cause of cancer deaths in developing countries. CC can be prevented through available preventive interventions. However, most patients in developing countries, such as Ethiopia, present late with advanced stage disease due to low participation in CC screening and require treatment involving multiple modalities. Women’s social, economic and cultural backgrounds have been associated with the level of participation in CC screening programmes. Therefore, this study aimed to assess women’s sexual autonomy as a determinant of lifetime CC screening among women in Addis Ababa, Ethiopia. An institutional-based case–control study was conducted in which controls were women who had received screening services during the last 5 years, and cases were randomly selected from women coming for other services but never screened or aware of the screening service. Accordingly, 294 women were enrolled. Data were collected by using a pre-tested standard questionnaire through interviewing. Bivariate and multivariable logistic regression analyses were performed to assess the women’s sexual autonomy as a determinant of lifetime CC screening. The study revealed higher sexual autonomy led to higher odds for having been screened (adjusted odds ratio (AOR) = 3.128, 95% CI (1.730, 5.658)). Moreover, direct referral to the screening service (AOR = 3.173, 95% CI (1.57, 6.45)) and parity had positively affected the lifetime uptake of CC screening (AOR = 2.844, 95% CI (1.344, 6.014)). We found that women’s own sexual autonomy was associated with the improvement of CC screening uptake. Empowering women could alleviate barriers to CC screening in the community.

## Introduction

Cervical cancer (CC) is the fourth most common cancer in women worldwide and the leading cause of cancer deaths in developing countries. Globally, an estimated 604,127 new cases and 341,831 deaths occur annually [1]. Moreover, 90% of the cases and most of the deaths occur in developing countries where most women remain undiagnosed and have limited or no access to treatment [2, 3]. CC incidence rates in sub-Saharan Africa, including Ethiopia, are the highest worldwide. It is the second leading cause of female cancer in Ethiopia [4]. In Ethiopia, about 7745 new CC cases and 5338 CC deaths are reported annually (5). In Ethiopia, the national CC screening coverage was very low (2.9%) in 2017 [6]. In Ethiopia, the screening coverage is very small compared to the WHO goal of 70% [7]. Most (80%) of the patients in Ethiopia are diagnosed late with advanced stage disease in which treatment may involve multiple modalities [8].

CC deaths and associated health care and social costs can be markedly reduced through appropriate screening and early detection interventions [2, 9]. The incidence and mortality of CC have been reduced in countries through organized screening programmes [10, 11]. In developing countries, including Ethiopia, the CC screening rate is low, ranging between 1 and 23% [12–14]. Recently, in Ethiopia, CC screening centres are being established to provide screening services for all eligible women. However, screening uptake in the community is still low [6, 15]. In Ethiopia, based on updated screening guideline, for the general population of women screening starting at age 30 and every 5–10 years of screening interval. For women living with HIV, screening starting at age 25 and every 3–5 years screening interval [16]. Individual, social and cultural factors have been associated with the CC screening uptake elsewhere [17–21]. Of individual determinants, women’s employment, educational level and household wealth index were reported to affect utilization of CC screening [17, 18]. Religious and cultural beliefs have also been associated with CC screening uptake [20–23]. Besides, women’s autonomy has been related to the participation level for CC screening [26]. The women’s autonomy could also be seen as a means to achieve gender equality as stipulated in the sustainable development goal (SDG). Studies have identified the positive effect of women’s autonomy for different health outcomes. In Nepal, a higher level of women's autonomy is reported to be associated with higher use of maternal health care service utilization [28]. In Lesotho, women with higher sexual autonomy had higher CC screening awareness than women with lower sexual autonomy [29]. In Kenya, higher uptake for the Pap test was found among women who had sexual autonomy when compared with their counterparts [26]. Low educational level, poor knowledge towards cervical cancer and screening, long distance to screening site, fear of positive result, embarrassment was some of the barriers of cervical cancer screening uptake in Ethiopia [12–15, 17–19]. Besides, data from other countries showing that greater sexual autonomy increase uptake [26]. In Ethiopia, there is one quantitative study describing sexual autonomy focussing on risky behaviour. A decline was noted [27]. In Ethiopia, studies addressed the socio-demographic, reproductive, clinical and health care-related determinants of CC screening uptake [12–14, 17–19, 30]. However, none of these studies examined the association between women’s autonomy and CC screening uptake. Thus, the aim of this study was to determine whether sexual autonomy determines lifetime CC screening uptake using a direct comparison.

## Material and methods

### Study setting, design and population

An institutional-based unmatched case–control study design was employed in Addis Ababa, Ethiopia. The study was conducted at five public health facilities (Saris family guidance clinic, Kolfe health center, Adisu Gebya health center, Zewditu hospital and Betsega MCH hospital). The catchment area roughly serves for more than one million populations. Women aged 30–49 years who were permanent residents of Addis Ababa were included. Cases were randomly selected women who were aware of the CC screening service and presenting for other services but never screened for CC. For the cases who had never received a screening before, we did not have the information weather they were not screened due to lack of offer or whether they had refused. Controls were women who had received and were aware of screening services in the past 3–5 years. Women who were severely ill during the data collection period, women who had undergone total hysterectomy and women who were not aware of CC screening services were excluded.

### Sample size and sampling procedures

The sample size was determined based on the 95% confidence level, 80% power, and 2 to 1 ratio of control to case. Accordingly, the sample size was 99 cases and 197 controls, including non-respondents, resulting in a total sample size of 296. Five out of 26 health facilities were selected randomly by the lottery method, based on flow of clients and current provision of CC screening services. The proportion of women selected from the health facilities was in accordance with proportional allocation. Every fifth client was selected to be interviewed. In case they were not aware of the service, the next person would be interviewed. In such away, the selection and interviewing continued until the required number of study participants was reached.

### Data collection procedures and measurements

Data on the socio-demographic and socioeconomic characteristics of women, reproductive variables (parity and sexual autonomy), wealth index, health and health system-related factors were collected using standard structured questionnaires [31] through face-to-face interviews by nine trained nurses who had been working at CC screening and treatment centres in Addis Ababa. Initially, the questioner was prepared in English and then translated to the local language (Amharic) for data collection. Then, the questionnaire further translated back to English to keep the consistency. The interviewing lasts 30 min for each respondent. The standard method was used to assess sexual autonomy. The tool we use was adapted from other source of study done on sexual autonomy and contraceptive use in Nigeria; It was chosen because it was very related to the cervical cancer screening uptake which is one of reproductive issue. Sexual autonomy was assessed by asking five yes or no types of questions: 1. Can you say ‘no’ to your husband/partner if you do not want to have sexual intercourse?; 2. In your opinion, is a husband justified in hitting or beating his wife if she refuses to have sex with him?; 3. Could you ask your husband/partner to use a condom if you wanted him to?; 4. If a wife knows her husband has a disease that she can contract during sexual intercourse, is she justified in asking him to use a condom when they have sex?; and 5. Is a woman justified in refusing sex if she is tired/not in the mood? [31].

The internal consistence of items was ensured by using cronbanches alpha coefficient. The coefficient value (α > 0.7) showed that the tool was reliable and appropriate to use in the study.

### Methods of analysis

Data were analysed by Stata SE 14 (64-bit). Principal component analysis (PCA) was used to construct a sexual autonomy index. Participants’ sexual autonomy having five components were analysed by PCA with STATA software, because sexual autonomy is not a single factor and consists of a composite of factors.

Applying principal component factors, eigen values were used to accept and drop the relevant factors. The eigen value range should be from greater than one to near zero. An eigenvalue of 1 means that the principal component would explain about one variable’s worth of the variability. Thus, the eigen value greater than 1 should be retained. Accordingly, two factors that had more than 1 eigen values were retained; the first factor was ‘decision of women for sexual intercourse’ and the second factor was ‘violating of women for refusing sex’. The second factor was selected because it had significant effect on cervical cancer screening uptake whereas the first factor did not have. Then, using one of these factors, STATA categorized the data into two levels of sexual autonomy: low and high.

Frequencies and percentages were produced to describe the socio-demographic characteristics of the study participants, and bivariate analysis of each variable with the outcome variable was done to produce a crude odds ratio (COR). Variables with a p-value < 0.25 were included in the multivariable logistic regression to assess the effect of independent variables with the outcome of interest by controlling the effect of covariates. The Hosmer–Lemeshow test was used to check the goodness of fit of the model. A p-value < 0.05 were used to decide the presence of statistical significance. The AOR and its respective 95% confidence interval were used to report variables found to have a statistically significant association with determinants of lifetime CC screening.

## Results

### Socio-demographic characteristics of the participants

A total of 98 (33.3%) cases and 196 (66.7%) controls were enrolled, with a 99% response rate. Among the study participants, 121 (61.7%) of the controls and 74 (75.5%) of the cases were found to be in the age group of 30–39 years old, while 75 (38.3%) of the controls and 24 (24.5%) of the cases were found in the age group of 40–49 years old. The mean and standard deviation of the age of controls and cases were 37.34 ± 6.75 years and 37.57 ± 5.56 years, respectively (Table 1).

**Table 1:** Socio-Demographic information of the study participants with respect to cervical cancer screening in Addis Ababa,2019

Variable	Controls		Cases		Total	
	N = 196	%	N = 98	%	N = 294	%
Age of the participants						
30–39 Years	121	61.73	74	75.51	195	66.3
40–49 Years	75	38.27	24	24.49	99	33.7
Mean (SD)	37.34 (6.75)	37.57 (5.56)	36.41 (6.50)			
Education level						
No formal education	29	14.80	15	15.31	44	14.97
Formal education	41	20.92	16	16.33	57	19.39
Primary	65	33.16	42	42.86	107	36.39
Secondary	57	29.08	23	23.47	80	27.21
College	4	2.04	2	2.04	6	2.04
Religion						
Orthodox	128	65.31	68	69.39	196	66.67
Islam	40	20.41	17	17.35	57	19.39
Protestant	21	10.71	11	11.22	32	10.88
Catholic	7	3.57	2	2.04	9	3.06
Occupation						
Housewife	43	21.94	36	36.73	79	26.87
Employee/private	83	42.35	38	38.78	121	41.16
Student	2	1.02	1	1.02	3	1.02
Merchant	41	20.92	17	17.35	58	19.73
Local drink seller	9	4.59	2	2.04	11	3.74
Daily laborer	5	2.55	2	2.04	7	2.38
Unemployed	13	6.63	2	2.04	15	5.10
Marital status						
Currently married	131	66.84	75	76.53	206	70.07
Separated	10	5.10	4	4.08	14	4.76
Divorced	15	7.65	7	7.14	22	7.48
Widowed	12	6.12	3	3.06	15	5.10
Unmarried	28	14.29	9	9.18	37	12.59

### Participants’ sexual autonomy

About 139 (70.90%) of the controls and 53 (54.08%) of the cases say no to husband/partner if not want to have sexual intercourse. On the other hand, about 19 (9.96%) of the controls and 15 (15.31%) of the cases their husband justified in hitting or beating his wife if she refuses to have sex with him (Table 2).

**Table 2:** Distribution of sexual autonomy questions response with respect to cervical cancer screening status in Addis Ababa, 2019

Variable		Screened		Non-Screened		Total	
		Freq	%	Freq	%	Freq	%
Say ‘No’ to husband/partner if not want to have sexual intercourse?	Yes	139	70.92	53	54.08	192	65.31
	No	57	29.08	45	45.92	102	34.69
Is a husband justified in hitting or beating his wife if she refuses to have sex with him?	Yes	19	9.69	15	15.31	34	11.56
	No	177	90.31	83	84.69	260	88.44
Ask your husband/ partner to use a condom if you wanted him to?	Yes	83	42.35	49	50.00	132	44.99
	No	113	57.65	49	50.00	162	55.10
If your husband has a disease that you can contract during sexual intercourse, are you justified in asking him to use a condom when you have sex?	Yes	162	82.65	53	54.08	215	73.13
	No	34	17.35	45	45.92	79	26.87
Refusing sex if you are tired/not in the mood?	Yes	156	79.59	49	50.00	205	69.73
	No	40	20.41	49	50.00	89	30.27

**Fig. 1:**
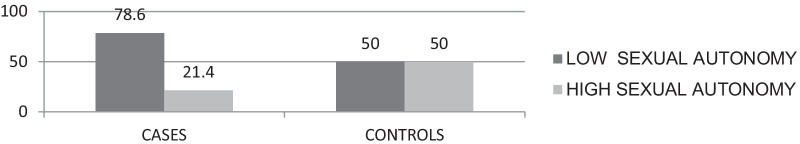
Participants’ sexual autonomy and cervical cancer screening in Addis Ababa, 2019

Pertaining sexual autonomy about 98 (50%) of the controls and 77(78.6%) of the cases had low sexual autonomy. On the other hand, about 98 (50%) of the controls and 21 (21.4%) of the cases had high sexual autonomy (Fig. 1).

### Health and health system-related factors

Concerning the health and health-related factors of CC, about 173 (88.20%) of the controls and 80 (81.63%) of the cases visited health facilities in the last 12 months. Likewise, 67 (34.10%) of the controls and 12 (12.24%) of the cases were referred from other health facilities. Similarly, about 52 (77.60%) of the controls and nine (75.00%) of the cases visited public health institutions in the past 12 months (Table 3).

**Table 3:** Health and health system related factors association with cervical screening in Addis Ababa, 2019

Variable		controls		Cases		Total	
		N = 196	%	N = 98	%	N = 294	%
Did you visit health facility in the last 12 month?	Yes	173	88.27	80	81.63	253	86.05
	No	23	11.73	18	18.37	41	13.95
Are you referred from other health facility?	Yes	67	34.18	12	12.24	79	26.87
	No	129	65.82	86	87.76	215	73.13
Which health institution have you visited?	Public	52	77.61	9	75.00	61	77.22
	Private	15	22.39	3	25.00	18	22.78
Does transportation provided for you from referring health institution?	Yes	6	8.96	1	8.33	7	8.86
	No	61	91.04	11	91.67	72	91.14
How long you stay to reach this health facility?	Mean	77.28		81.2		77.8	
	SD	52.900		36.4		50.5	

### Maternal health-related factors

The majority of the women, which is 107 (54.5%) of the controls and 52 (53.06%) of the cases, became pregnant 2–5 times in the past 5 years. In addition to this, about 158 (80.6%) of the controls and 67 (68.37%) of the cases gave birth 1–4 times. Similarly, 157 (80.1%) of the controls and 70 (71.14%) of the cases currently have 1–4 children (Table 4).

**Table 4:** Maternal health related factors with respect to cervical cancer screening in Addis Ababa, 2019

Variable		Controls		Cases		Total	
		N = 196	%	N = 98	%	N = 294	%
How many times you become pregnant (number of pregnancy)?	1 time	64	32.65	36	36.73	100	34.01
	2–5 times	107	54.59	52	53.06	159	54.08
	> 5 times	25	12.76	10	10.20	35	11.90
How many times you gave birth (parity)?	0	18	9.18	23	23.47	41	13.95
	1–4	158	80.61	67	68.37	225	76.53
	> 4	20	10.20	8	8.16	28	9.52
How many children did you have?	0	20	10.20	21	21.43	41	13.95
	1–4	157	80.10	70	71.43	227	77.21
	> 4	19	9.69	7	7.14	26	8.84

### Determinants of cervical cancer screening uptake among women

In bivariate analyses variables such as parity, referral from another health facility, and sexual autonomy, the ages of respondents and partners were candidates for multivariable logistic regression analysis. In the final regression model, sexual autonomy, referral from another health facility and parity were statistically significant determinants for lifetime CC screening among women, with a 95% confidence level. However, monthly income and the occupation of the participant failed to be significant in the multivariable logistic regression model (Table 5).

**Table 5:** Multivariable logistic regression analyses for determinants of cervical cancer screening among women in Addis Ababa, 2019

Variables	Screening status		COR (95%C.I)	AOR (95% C.I)
	Controls (N = 196)	Cases (N = 98)		
Age of the participants				
30–39 years	121(61.73%) (61.73.%)	74 (75.51%)	1	1
40–49 years	75 (38.27%)	24 (24.49%)	1.91 (1.11, 3.29)*	0.728 (0.299,1.77)
Age of the partners				
30–39 years	80(40.82%) (61.73.%)	52 (53.06%)	1	1
40–49 years	65 (33.16%)	35 (35.71%)	1.207 (0.704, 2.070)	0.859 (0.445, 1.659)
> 50 years	51 (26.02%)	11 (11.22%)	3.014 (1.439, 6.311)*	2.763 (0.817, 9.340)
Sexual autonomy				
Low	98(50%) (61.73.%)	77 (78.57%)	1	1
High	98(50%)	21 (21.43%)	3.667(2.100, 6.405)*	3.128 (1.73, 5.658)*
Referred from other HF				
No	129 (65.82%)	86(87.76%)	1	1
Yes	67 (34.18%)	12 (12.24%)	3.72(1.90,7.30)*	3.173 (1.57, 6.45)*
How many times gave birth(parity)				
0	18(9.18%)	23(23.47%)	1	1**
1–4	158(80.61%)	67(68.37%)	3.013(1.527, 5.946)*	2.844 (1.344, 6.014)*
> 4	20(10.20%)	8(8.16%)	3.194(1.450, 8.912)*	1.617 (0.436, 5.992)

^*^P < 0.05; CI: Confidence interval, COR: Crude odd ratio, AOR: Adjusted odd ratio for age, sexual autonomy, referred from HF and parity

The odds of low sexual autonomy were 3.128 times greater in women who were aware of CC screening but had not screened before (cases) compared to women who had been screened in the past 3–5 years (controls) (AOR = 3.128, 95% CI (1.730, 5.658)).

The odds of not being referred from other health facilities for screening services were 3.173 times greater in women who were aware of CC screening but had not screened before(cases) compared to women who had been screened in the past 3–5 years(controls) (AOR = 3.173, 95% CI (1.57, 6.45)), and the odds of parity 0 were 2.844 times higher in women who were aware of CC screening but had not screened before(cases) compared to women who had been screened in the past 3–5 years(controls) (AOR = 2.844, 95% CI (1.344, 6.014)).

## Discussion

This study examined if women’s autonomy affects the lifetime CC screening uptake among women who were aware of the service before and were receiving other health services at five health facilities in Addis Ababa.

The study revealed higher sexual autonomy associated to higher odds for having been screened for CC. This finding is similar with a study conducted in Kenya [26]. This implies that women’s sexual autonomy plays a crucial role in lifetime CC screening uptake. It could be that women with more sexual autonomy tend to engage more with preventive health care services, such as CC screening, for their own sexual and reproductive health. Furthermore, the fact that women with higher sexual autonomy have higher health-seeking behaviour might explain our study finding. This is supported by the current study that more than half (55.3%) of women who did not visit health facility had lower sexual autonomy. Moreover, women who visited health facility were 1.5 times more likely to have higher sexual autonomy than women who did not visit the health facility. This is consistent with a study done in Ethiopia [32]. This is also supported by another study that found more autonomous women are more likely to visit a health facility for health care service than their counterparts [33]. Interventions such as increasing women’s education [34, 35] and raising awareness about women’s sexual right [36, 37] have been effective to improve women sexual autonomy elsewhere in the world which might be benefit to be implemented in Ethiopia. Thus, the existing national policies should consider these interventions as a means to improve women sexual autonomy. However, the finding of this study is not consistent with the study conducted in Lesotho that found women’s sexual autonomy was associated with CC screening awareness not with action [29]. The reason for this difference might be the difference in study design used. The study done in Lesotho used a cross-sectional study design, which is limited in showing casual association between women’s sexual autonomy and CC screening uptake. Although not statistically significant, there were some differences in the demographics between the cases and controls. For instance, higher sexual autonomy was observed among unmarried women (41%) than married women. This could be unmarried women has the right to refuse sexual intercourse unlike married women who might not have that power to refuse intercourse from husband/partner.

Besides women’s sexual autonomy as a predictor of CC screening uptake, direct referral for the service and parity were other predictive variables we found in our study. Those women who were referred from other health facilities for the service were more likely to be screened than their counterparts. This is consistent with a similar study that reported those women who visited health facilities were more likely to have increased CC screening uptake [29]. It might be that those referred for the service might be told about the benefits and risks of not having CC screening. In terms of parity, women with a number of births were more likely to have been screened than their counterparts. This could be due to their previous attachments with health facilities, which could expose them to reproductive health services information, including the need to take part in CC screening services. This is consistent with studies conducted in China [34].

We acknowledge the following potential limitations of the study that affect the results in some ways. First, there was no comparable data from Ethiopia so therefore we simply focussed on differences according to higher levels of sexual autonomy. Second, due to the retrospective nature of data collection, the recall bias may have affected the results in both groups. However, we have attempted to mitigate this by giving more time and asking related questions to triangulate. Second, some of the study participants may have been shy responding to somewhat sensitive and culturally taboo questions we used to measure the women’s autonomy. However, we used only female interviewers to reduce such social desirability bias. This study had the following strength: a study on the effect of women’s sexual autonomy on CC screening uptake is directly related to SDG3, which has wider policy and strategy implications in Ethiopia.

## Conclusion

We found that the sexual autonomy of women significantly predicted the improvement of lifetime CC screening uptake. This study suggests a broad-based strategy to endeavour empowerment of women in various ways so as to improve the uptake of women for CC screening.

## Data Availability

The data sets used and/or analyzed during the current study are available from the corresponding author on reasonable request.

## Abbreviations

AOR: Adjusted odds ratio
CC: Cervical cancer
CI: Confidence interval
COR: Crude odds ratio
EMOH: Ethiopian Ministry of Health
HF: Health facility
PCA: Principal component analysis
SD: Standard deviation
VIA: Visual inspection with acidic acid
WHO: World Health Organization
